# Climate Change as a Chronic Stressor: Biological and Psychological Pathways Linking Environmental Change to Human Well-Being

**DOI:** 10.3390/life16101763

**Published:** 2026-10-21

**Authors:** Giuseppe Marano, Camilla Scialpi, Antonio Sottile, Oksana Di Giacomi, Marco Lanzetta, Gianandrea Traversi, Osvaldo Mazza, Esmeralda Capristo, Eleonora Gaetani, Marianna Mazza

**Affiliations:** 1 Department of Neuroscience, Head-Neck and Chest, Section of Psychiatry, Fondazione Policlinico Universitario Agostino Gemelli IRCCS, Largo Agostino Gemelli 8, 00168 Rome, Italy; camilla.scialpi@libero.it (C.S.);; 2 Department of Neuroscience, Section of Psychiatry, Università Cattolica del Sacro Cuore, Largo Agostino Gemelli 8, 00168 Rome, Italy; 3 Unit of Medical Genetics, Department of Laboratory Medicine, Ospedale Isola Tiberina-Gemelli Isola, 00186 Rome, Italy; gianandrea.traversi@gmail.com; 4 Spine Surgery Department, Bambino Gesù Children’s Hospital IRCCS, 00168 Rome, Italy; osvaldo.mazza1973@hotmail.it; 5 Department of Translational Medicine and Surgery, Università Cattolica del Sacro Cuore, 00168 Rome, Italyeleonora.gaetani@unicatt.it (E.G.); 6 Unit of Internal Medicine, Cristo Re Hospital, 00167 Rome, Italy

**Keywords:** climate change, chronic stress, mental health, eco-anxiety, ecological grief, heat exposure, inflammation, neuroplasticity, health inequalities

## Abstract

Climate-related hazards are associated with adverse mental and physical health outcomes, but exposure-specific human findings are often discussed together with general stress mechanisms without clearly distinguishing their evidentiary strength. This narrative review addresses that gap by examining climate change as a context for recurrent thermal, disaster-related, and psychosocial stress, rather than as a single measurable exposure. Evidence is organized around three interacting biological domains: neuroendocrine regulation, inflammation and oxidative stress, and neuroplasticity. Human observational studies support associations between heat, extreme events, and mental health outcomes; short-term human experiments and animal models provide mechanistic insights but do not establish a chronic biological mediation pathway from climate change to psychiatric illness. Indirect pathways include displacement, livelihood disruption, loss, and anticipatory distress. Air pollution is treated as an interacting exposure with both climate-related and independent determinants. Eco-anxiety, climate anxiety, eco-distress, and ecological grief are distinguished from diagnosable disorders, with clinical relevance assessed through functional impairment and individual context. The review’s contribution is an evidence-qualified framework linking exposure timing, biological plausibility, psychosocial appraisal, and unequal adaptive capacity. Practical implications include contextual mental health assessment, continuity of psychiatric care during heat and disasters, and incorporation of psychosocial support into adaptation planning. The proposed framework remains hypothesis-generating and requires prospective testing with repeated exposure, biomarker, and clinical assessments.

## 1. Introduction

Climate change is now recognized as one of the major global public health emergencies. Beyond its widely acknowledged consequences for ecosystems and physical health, an increasing body of scientific literature highlights its impact on mental health and psychological well-being. Rising global temperatures, the growing frequency and intensity of extreme weather events, and environmental degradation have been associated with increased psychological distress and higher rates of mental disorders across diverse populations [1,2,3].

The health effects of climate change occur through multiple pathways, including heat stress, the increased spread of infectious diseases, exposure to extreme weather events, and reduced agricultural production and the consequent risk of malnutrition [4]. Within this framework, heat stress represents a particularly relevant factor, as available evidence indicates its association with increased emergency department visits and hospitalizations [5], adverse pregnancy outcomes, and increased cardiovascular, respiratory, and all-cause mortality, particularly among older and vulnerable populations [6].

Climatic conditions affect human well-being through both direct and indirect mechanisms. Acute events, such as heatwaves, floods, and wildfires, can trigger immediate stress responses; at the same time, persistent environmental changes, such as prolonged exposure to elevated temperatures and ecosystem instability, are associated with long-term psychological and physiological stress. Importantly, evidence suggests that mental health effects may also emerge in individuals not directly exposed to extreme events, reflecting broader psychosocial responses to environmental change [7].

Epidemiological reviews report associations between high ambient temperatures or heatwaves and mental health-related morbidity and mortality. The outcomes and magnitude vary across studies, and observed associations should not be interpreted as evidence that heat produces a specific psychiatric disorder in every exposed person [8,9].

Controlled human heat-exposure studies provide evidence of short-term physiological and psychological responses, whereas animal experiments examine neural injury under exposure conditions that may differ substantially from everyday ambient heat [3,10,11,12]. These evidence streams should not be treated as interchangeable demonstrations of chronic brain damage in climate-exposed populations.

A small randomized crossover study in humans reported acute anxiety and blood biomarker changes after experimental heat exposure [3]. Heat-exposed animal models also suggest inflammatory and neural effects [11,12]. These findings provide mechanistic plausibility; they do not establish that recurrent ambient heat causes persistent HPA axis dysregulation or psychiatric disorders in humans.

Here, a chronic stressor denotes a sustained or recurrent demand on adaptive systems, rather than a psychiatric diagnosis. Climate change provides a long-term context in which some stressors recur or persist; individual heatwaves and disasters may still be acute exposures. Whether these exposures produce chronic stress depends on duration, recurrence, recovery, threat appraisal, and access to protective resources [13,14].

Terminology is used consistently throughout. Eco-distress is the broad umbrella for emotional, cognitive, and behavioral distress related to environmental degradation or threat. Eco-anxiety refers more specifically to anxiety-related responses to ecological threats, while climate anxiety concerns climate change in particular. Ecological grief denotes grief connected to experienced or anticipated loss of species, ecosystems, or meaningful landscapes. These constructs can overlap, but none by itself denotes an anxiety, depressive, or grief disorder; diagnostic assessment requires established criteria and contextual evaluation [15,16,17,18,19].

The review focuses on the intersection of climate-related exposures, stress biology, and mental health, with physical comorbidity considered where it modifies psychiatric vulnerability or care. Its specific contribution is to separate exposure-associated clinical findings from experimentally supported mechanisms and broader stress hypotheses. Existing studies describe climate-related mental health pathways [2,13], heat mechanisms [20], and climate-related emotional responses [21,22]; the present synthesis compares these levels within a single, explicitly hypothesis-generating multisystem framework.

## 2. Materials and Methods

This article is a narrative review with a thematic, non-exhaustive synthesis, rather than a systematic review or meta-analysis. Literature searches were conducted in PubMed, Scopus, and Web of Science Core Collection. The last literature search was conducted on 1 July 2026. The principal emphasis was contemporary literature, with earlier foundational publications considered for mechanistic context; no uniform publication-year restriction was applied.

Selection prioritized original human studies and systematic reviews addressing measured heat, disaster, drought, or climate-related psychosocial exposures and mental health outcomes. Experimental human and animal studies were considered for biological plausibility, while general stress-neuroscience studies were explicitly treated as indirect evidence. Narrative reviews and institutional guidance provided conceptual and policy context and were not treated as primary empirical studies. English-language publications were considered. Articles without relevance to the exposure–stress–mental health question were excluded from the thematic synthesis.

The following database-specific search strategies were used.

PubMed:

(“Climate Change”[Mesh] OR “Hot Temperature”[Mesh] OR “climate change”[tiab] OR “global warming”[tiab] OR “ambient temperature”[tiab] OR “heat stress”[tiab] OR heatwave*[tiab] OR “heat wave”[tiab] OR “extreme weather”[tiab] OR flood*[tiab] OR wildfire*[tiab] OR drought*[tiab] OR “eco-anxiety”[tiab] OR “climate anxiety”[tiab] OR “eco-distress”[tiab] OR “ecological grief”[tiab]) AND (“Mental Health”[Mesh] OR “Mental Disorders”[Mesh] OR “mental health”[tiab] OR psychiatr*[tiab] OR depress*[tiab] OR anxi*[tiab] OR PTSD[tiab] OR suicid*[tiab] OR “psychological distress”[tiab] OR wellbeing[tiab] OR “well-being”[tiab] OR neuroplastic*[tiab] OR neuroendocrin*[tiab] OR “HPA axis”[tiab] OR cortisol[tiab] OR inflamm*[tiab] OR “oxidative stress”[tiab] OR BDNF[tiab] OR multimorbid*[tiab]) AND english[lang]

Scopus:

TITLE-ABS-KEY((“climate change” OR “global warming” OR “ambient temperature” OR “heat stress” OR heatwave* OR “heat wave*” OR “extreme weather” OR flood* OR wildfire* OR drought* OR “eco-anxiety” OR “climate anxiety” OR “eco-distress” OR “ecological grief”) AND (“mental health” OR psychiatr* OR depress* OR anxi* OR PTSD OR “post-traumatic stress” OR suicid* OR “psychological distress” OR wellbeing OR “well-being” OR neuroplastic* OR neuroendocrin* OR “HPA axis” OR cortisol OR inflamm* OR “oxidative stress” OR BDNF OR multimorbid*)) AND LIMIT-TO(LANGUAGE, ”English”)

Web of Science Core Collection:

TS = ((“climate change” OR “global warming” OR “ambient temperature” OR “heat stress” OR heatwave* OR “heat wave*” OR “extreme weather” OR flood* OR wildfire* OR drought* OR “eco-anxiety” OR “climate anxiety” OR “eco-distress” OR “ecological grief”) AND (“mental health” OR psychiatr* OR depress* OR anxi* OR PTSD OR “post-traumatic stress” OR suicid* OR “psychological distress” OR wellbeing OR “well-being” OR neuroplastic* OR neuroendocrin* OR “HPA axis” OR cortisol OR inflamm* OR “oxidative stress” OR BDNF OR multimorbid*)) AND LA = (English)

These strategies combined climate-related hazards or environmental distress with mental health outcomes and the biological domains discussed in the review. Verified database-specific retrieval totals and counts at the identification, deduplication, screening, and inclusion stages are not available in the retained documentation. Accordingly, no screening denominator, exclusion counts, PRISMA flow diagram, independent duplicate screening, or exhaustive coverage is claimed. The number of references in the bibliography should not be interpreted as the number of eligible primary studies. These reporting limitations are considered in Section 9.2.

The evidence was synthesized by exposure route and by three biological domains: neuroendocrine regulation, inflammation and oxidative stress, and neuroplasticity. Study design, population or experimental model, outcome ascertainment, main findings, and important limitations were compared for representative evidence in Table 1. Clinical associations, experimental effects, and general stress hypotheses were kept distinct. No pooled effect estimate or formal risk-of-bias score was generated. Figure 1 presents the author-proposed conceptual framework, rather than a validated causal model.

## 3. Exposure Pathways and Climate-Related Stress

Climate change refers to long-term shifts in climatic conditions and hazard probabilities; meteorological exposures are the temperature, humidity, duration of heat, or extreme events actually experienced by a person or population. A weather–health association does not itself establish the proportion attributable to anthropogenic climate change. Following Palinkas and Wong [13], acute events, slower changes such as drought, and long-term environmental transformation are considered separately. Direct pathways involve thermal strain, injury, or disaster exposure. Indirect psychosocial pathways include livelihood loss, displacement, food or housing insecurity, disruption of care, and perceived future threat. Socioeconomic conditions can precede exposure, modify its effects, or arise as consequences; their analytical role depends on timing and the causal question [4,13].

Disaster-exposed populations show associations with PTSD, anxiety, depressive symptoms, and impaired quality of life; longitudinal studies indicate that some symptoms persist, although trajectories differ by exposure and post-disaster resources [23,24,25,26,27,28,29,30,31]. Heat-related mental health morbidity and mortality associations are reported across epidemiological studies, but temperature definitions, lag periods, diagnoses, and contextual adjustment vary [8,9,32]. Drought-related distress is particularly relevant where livelihoods depend on agriculture; financial strain and uncertainty may contribute, and confounding by pre-existing rural disadvantage remains important [33,34,35,36,37,38,39]. These findings support exposure-specific vulnerability rather than a single inevitable clinical sequence.

Air pollution is an interacting environmental exposure, not a synonym for climate change. Wildfire smoke and climate-sensitive ozone can be linked to climatic conditions, while traffic and industrial pollution also have independent sources. Pollutants may act as co-exposures, mediators in a specified pathway, or confounders of temperature–health associations. Urban form, deprivation, seasonality, occupation, and behavioral adaptation can further influence both exposure and outcome. Studies should therefore specify which exposures are measured and which relationships are being estimated [40,41,42].

Pihkala’s taxonomy emphasizes that climate-related emotions include worry, grief, anger, and other responses, not anxiety alone [15]. Solastalgia concerns distress associated with unwelcome change to a familiar place; ecological grief concerns environmental loss. Neither response should be equated automatically with psychopathology [13,16]. Meteorosensitivity, assessed in instruments such as METEO-Q, concerns responses to weather variation and is conceptually distinct from climate anxiety or ecological grief; meteorosensitivity measurement does not establish climate-specific validation [19].

Youth surveys document climate-related concern and perceived inadequacy of institutional responses, but concern prevalence is not a prevalence estimate for anxiety disorders [43]. Threat appraisal, fairness, attachment to place, and collective efficacy may shape emotional responses independently of direct hazard exposure.

## 4. Three Biological Domains and Their Evidentiary Limits

Three biological domains organize the mechanistic discussion: neuroendocrine regulation; inflammation and oxidative stress; and neuroplasticity. Human heat experiments, animal exposure studies, and general stress research address different questions. Within each domain, evidence is assessed for its directness to measured climate-related exposure, consistency across designs, and transferability to persistent human clinical outcomes. Representative study-level comparisons are provided in Table 1.

### 4.1. Neuroendocrine Regulation

The HPA axis regulates glucocorticoid secretion and interacts with autonomic, metabolic, and immune responses to stress [44,45]. Heat can impose acute thermoregulatory and metabolic demands, but transient HPA activation is an adaptive response and should not be labeled dysregulation without evidence of altered basal activity, feedback, recovery, or persistent exposure-related change.

In the established stress-response pathway, hypothalamic corticotropin-releasing hormone (CRH) stimulates pituitary adrenocorticotropic hormone (ACTH), followed by adrenal glucocorticoid secretion. Cortisol influences energy mobilization, immune activity, and emotional and cognitive processes [44,45]. This physiology provides a plausible basis for heat-stress research; it does not by itself demonstrate climate-specific endocrine pathology.

Arginine vasopressin can potentiate CRH-related ACTH secretion, while hydration and osmotic regulation are relevant to thermal stress [44,45]. In Fang et al.’s randomized crossover study of 20 college students, 1.5 h of experimental heat exposure was associated with increased anxiety and peripheral neuroendocrine, inflammatory, and metabolic changes [3]. The controlled design supports an acute exposure effect in that setting, but the sample and exposure window limit extrapolation to chronic psychiatric vulnerability.

Repeated glucocorticoid signaling and impaired recovery are established concerns in general chronic-stress research [14]. However, the proposed progression from recurrent climate-related exposures to glucocorticoid resistance or sustained psychiatric vulnerability remains insufficiently tested in longitudinal human studies. Findings depend on exposure intensity, acclimatization, circadian sampling, medication, sleep, and concurrent stressors; consistency of a persistent HPA phenotype in climate-exposed populations is not established [20,44,45].

### 4.2. Inflammation and Oxidative Stress

Inflammatory and redox processes are discussed together because they interact biologically and are often assessed within the same experimental studies. Heat-exposed mice show neuroinflammatory and memory-related changes [12], while a short-term human experiment reported peripheral changes compatible with inflammatory and oxidative responses [3]. The direction of these findings is mechanistically coherent, but their magnitude and clinical significance cannot be assumed to be uniform across species or everyday exposure settings.

Heat-related tissue stress can engage danger-signal and innate immune pathways; the DAMP framework provides general mechanistic context rather than direct proof of a chronic climate-related inflammatory syndrome [46,47]. Pulmonary and systemic responses to particulate pollution represent a distinct, partially overlapping exposure pathway [40,41].

Oxidative stress reflects an imbalance between reactive species and antioxidant defenses. General neuroscience research supports interactions with mitochondrial function and neural signaling [48]. Experimental thermal exposures can test these processes, but the severity of laboratory hyperthermia and animal conditions must be considered before interpreting them as evidence of long-term ambient-temperature effects in humans [12,49].

Pollution and heat may occur together, but a synergistic interaction should be claimed only when jointly measured exposures and an explicit interaction analysis support it. Biological plausibility alone cannot establish that their combined clinical effect exceeds additive or multiplicative expectations [40,41,42].

Peripheral cytokines, glutathione-related measures, and metabolomic signals are candidate research indicators rather than validated biomarkers of climate-related psychiatric illness [3,48]. Blood measures do not establish neuroinflammation, central neurotransmitter concentrations, or causal mediation. Their interpretation requires repeated sampling, control of circadian timing, infection, smoking, medication and adiposity, and appropriate statistical handling of multiple comparisons.

### 4.3. Neuroplasticity and Neural Vulnerability

Neuroplasticity offers a plausible bridge between stress regulation and cognitive or emotional adaptation, but much of the relevant evidence comes from general stress models rather than climate-specific research [14,50]. This distinction limits claims about chronic environmental exposure.

Neuroplasticity, the capacity of the nervous system to modify synaptic structure and function, is sensitive to stress intensity, duration, and recovery. Experimental studies of chronic stress support dendritic and attentional changes, but do not establish identical effects from recurrent ambient heat in humans [14,50].

Animal and cellular research suggests that glucocorticoid exposure, inflammation, and oxidative processes can alter synaptic structure and function [14,48,50]. Heat-exposed mouse studies also report neural and cognitive effects [11,12]. These observations support biological plausibility while leaving the dose–response relationship, reversibility, and relevance to human psychiatric diagnoses uncertain.

BDNF supports neuronal and synaptic function, and corticosterone experiments in rats demonstrate region-specific reductions in BDNF expression [51]. BDNF-related regulation of GABAergic transmission provides additional general mechanistic context [52]. Neither finding establishes a persistent BDNF deficit in people exposed to climate-related hazards.

Peripheral GABA- and BDNF-related changes in a short-term heat experiment should not be interpreted as direct measurements of brain GABAergic tone or synaptic plasticity [3]. A blood-based signal may differ from central signaling because of tissue sources, compartmentalization, and assay properties.

Across this domain, experimental findings are compatible with neural vulnerability under selected stress conditions, but longitudinal human evidence connecting repeated exposure, measured neural change, and subsequent psychiatric illness remains limited. Claims of accelerated neurodegeneration or an inevitable erosion of neural resilience therefore exceed the available evidence [14,20].

Table 1 compares representative exposure-specific and general mechanistic studies. It distinguishes study-level outcomes from proposed pathways, identifies major limitations, and prevents observations in animals or peripheral biomarkers from being read as confirmed human clinical mechanisms.

**Table 1:** Representative evidence for climate-related exposures, mental and physical outcomes, and stress mechanisms.

Study and Design	Exposure	Population or Model	Principal Outcome	Main Finding	Major Limitation
Fang et al., 2023 [3]; randomized crossover experiment	1.5 h laboratory heat intervention	20 college students in Beijing	Acute anxiety; blood neuroendocrine and multi-omic measures	Increased anxiety with peripheral stress-related changes	Small, young sample; acute exposure; blood findings do not establish central or chronic pathology
Lee et al., 2015 [12]; animal experiment	Experimental heat exposure	Mice	Memory performance; neuroinflammatory measures	Heat exposure associated with memory impairment and neuroinflammation	Species and exposure transferability; not a human psychiatric endpoint
Liston et al., 2006 [50]; general stress experiment	Experimental stress, not a climatic exposure	Rats	Prefrontal dendritic morphology and attentional set-shifting	Stress-related morphology associated with attentional impairment	Indirect mechanistic context; cannot establish a climate-specific pathway
Liu et al., 2021 [8]; systematic review and meta-analysis	High ambient temperature and heatwaves	Human epidemiological studies	Mental health-related morbidity and mortality	Per 1 °C: morbidity RR 1.009 (95% CI 1.007–1.015); mortality RR 1.022 (1.015–1.029)	Observational studies; varying exposures, lag periods and outcomes; pooled estimates are not individual causal effects
Hickman et al., 2021 [43]; cross-sectional survey	Climate concern and perceived government response	10,000 participants aged 16–25 across 10 countries	Self-reported climate emotions and daily life interference	Climate concern and negative appraisals of government responses were reported	Cross-sectional self-report; no diagnostic prevalence or temporal inference
Cosh et al., 2024 [21]; systematic review	Measured eco-anxiety	Adult quantitative studies	Distress and affective symptom measures	Associations with psychological burden, varying across definitions	Predominantly correlational evidence; heterogeneous measurement and ascertainment
Gebhardt et al., 2026 [22]; systematic review with meta-analysis	Eco-distress measures	Adult questionnaire studies	Correlations with mental health measures	Positive associations with substantial between-study heterogeneity	Cannot infer individual diagnosis or a universal cutoff; psychometric reporting incomplete
Kazi et al., 2024 [42]; systematic review	Temperature, ozone, wildfire smoke and extreme events	492 observational studies	Cardiovascular morbidity and mortality	Evidence varied by exposure; more consistent for temperatures, ozone and selected extreme events	Heterogeneous observational designs; some exposures and lower-income settings underrepresented
Tang et al., 2025 [53]; longitudinal cohort	Heatwaves and cold spells	9475 CHARLS participants aged ≥ 45 years	Incident multimorbidity	Positive associations across temperature-event definitions	Residual confounding; residential exposure estimates and disease ascertainment; observational mediation not established

Note. This is a selective evidence comparison, not an exhaustive study inventory or a formal certainty-of-evidence assessment. Primary studies and evidence syntheses are identified separately. General stress experiments are indirect evidence for the climate-related framework. Abbreviations: CHARLS, China Health and Retirement Longitudinal Study; CI, confidence interval; RR, risk ratio.

## 5. Psychological Responses and Mental Health Outcomes

Climate-related exposure and environmental threat appraisal are associated with both subclinical distress and diagnosable disorders. Disaster-related PTSD, heat-associated clinical events, and anticipatory climate concern should be distinguished because their exposures, ascertainment, and clinical implications differ [2,8,13]. A dimensional description of distress complements rather than replaces disorder-specific assessment.

Persistent disruption, loss, and inadequate recovery may be associated with reduced well-being, particularly alongside socioeconomic disadvantage or pre-existing illness. However, distress trajectories are heterogeneous and may include adaptation, resilience, delayed symptoms, or recovery. A progressive deterioration should not be presumed for every exposed individual [13,27,54].

Eco-anxiety and broader eco-distress are correlated with anxiety, depressive symptoms, and poorer well-being in quantitative studies, but these constructs are not interchangeable with psychiatric diagnoses [21,22]. Youth concern may also reflect perceived injustice and inadequate institutional action [43]. Cross-sectional self-report findings cannot determine whether climate-related distress precedes clinical symptoms, follows them, or shares underlying social and psychological determinants.

Biological susceptibility and distress may interact, but a reciprocal feedback loop remains a model assumption unless tested with repeated measurements and appropriate temporal analysis. General stress biology makes such interactions plausible; climate-specific clinical mediation has not been established [14,20].

The distinction between proportionate concern and clinically significant impairment requires attention to sleep, daily functioning, persistence, avoidance, safety, and coexisting disorders. Environmental awareness, activism, or ecological grief alone should not be treated as evidence of pathology. Protective factors and cultural meaning should be assessed alongside vulnerability [15,16,21].

## 6. Physical Comorbidity and Psychiatric Vulnerability

Physical outcomes are considered here because thermal strain, cardiometabolic illness, and disrupted care may complicate psychiatric assessment and treatment. The concept of multimorbidity describes coexisting conditions; it does not demonstrate that they share a single climate-induced biological cause. Environmental and social exposures should be specified rather than collapsed into an undifferentiated stress burden [42,53].

### 6.1. Cardiovascular and Other Physical Outcomes

Kazi et al.’s systematic review included 492 observational studies of climate-related environmental exposures and cardiovascular outcomes. Evidence was more consistent for extreme temperatures, ozone, and some extreme events than for wildfire smoke or drought. Heterogeneous designs and predominantly observational data limit causal interpretation and extrapolation across settings [42].

Thermal strain can affect hydration, vascular responses, and cardiovascular load, while cold exposure imposes different physiological demands [10,42]. These established acute responses should be distinguished from the hypothesis that recurrent exposure produces cumulative allostatic burden relevant to psychiatric illness.

Air pollution has independent cardiopulmonary pathways and can accompany climate-sensitive hazards. Its effects should therefore be assessed separately and jointly with temperature where data allow; a pollutant-health association is not automatically a climate-change effect [40,41,42].

A systematic review of heat exposure in low- and middle-income countries examined cardiovascular, respiratory, and diabetes-related outcomes [55]. Such evidence is relevant to care planning but does not establish heat-induced insulin resistance or a common metabolic mechanism across psychiatric disorders.

Reviews of climate-related immune effects identify multiple environmental pathways, including thermal exposure, pollutants, allergens, and infection-related changes [56]. These are not equivalent to a validated inflammatory phenotype of climate-related psychological distress.

Table 2 translates exposure, susceptibility, and access barriers into practical assessment and preparedness priorities. It complements the study-level evidence comparison in Table 1 and is intended as an author-proposed clinical and planning aid, not a validated risk classification.

Figure 2 distinguishes the physical comorbidity domain from the overarching exposure framework in Figure 1 and the preparedness framework in Figure 3. Metabolic dysregulation is shown as an interacting susceptibility factor or proposed intermediate process, rather than a meteorological exposure.

### 6.2. Mental–Physical Health and Continuity of Care

Mental and physical illness may interact through behavior, medication, sleep, and access to care, as well as through general stress pathways. During heat or disasters, impaired hydration, acute medical illness, and interrupted treatment can complicate psychiatric presentations [2,54]. These care-related pathways can be clinically important without proof of a chronic endocrine mediation mechanism.

Housing disruption, financial strain, and inaccessible services can interfere with medication adherence and disease self-management. Their temporal role should be assessed: these factors may be pre-existing confounders, exposure-related mediators, or effect modifiers depending on the study question [13,54].

A CHARLS cohort analysis of 9475 adults aged at least 45 years associated heatwaves and cold spells with incident multimorbidity, defined as at least two chronic diseases [53]. The study is observational and uses environmental and disease ascertainment that may not fully capture individual exposure or clinical complexity. It supports an association, not a proven biological feedback spiral or accelerated functional decline.

Allostatic load provides a useful hypothesis about the cost of recurrent adaptation [14]. The hypothesis becomes testable only when exposure history, recovery, repeated biological measures, and clinical outcomes are specified. It should not be inferred merely from co-occurring mental and physical conditions.

## 7. Susceptibility and Unequal Adaptive Capacity

Climate-health vulnerability arises from exposure, sensitivity, and adaptive capacity. These dimensions vary within as well as between demographic groups; chronological age, diagnosis, or income alone cannot capture individual risk [54,57].

### 7.1. Socioeconomic Disadvantage and Climate Vulnerability

Socioeconomic disadvantage may affect residential and occupational exposure, baseline disease burden, and access to cooling, transport, healthcare, and social support. It is therefore both a determinant of exposure and a potential modifier of health associations, rather than a single interchangeable covariate [57].

In urban settings, heat, pollutants, housing conditions, and access to green space can cluster. Analyses must consider neighborhood conditions and individual behavior to avoid assigning effects of deprivation or pollution to temperature alone. Adaptation measures should also be assessed for affordability and accessibility, rather than assuming equal benefit [57,58].

Cooling access, safe housing, and green infrastructure should be accompanied by measures that protect disadvantaged residents from exclusion or displacement. The planning implication is to monitor who receives adaptation benefits and who faces continuing exposure, using locally appropriate indicators [57,59].

### 7.2. Aging, Childhood, and Pre-Existing Mental Disorders

Older adults may have reduced thermoregulatory reserve, multimorbidity, polypharmacy, or limited support. Heat-related dehydration and acute medical illness can present with cognitive or psychiatric symptoms, requiring integrated assessment rather than automatic attribution to a mental disorder [5,54].

Medication-related risk requires individualized review. Antipsychotics and anticholinergic burden may impair thermoregulation; heat-related dehydration can also complicate lithium safety. Medication changes should be clinician-led and based on the person’s treatment, medical status, hydration, and exposure, rather than routine discontinuation during hot weather [54].

Reviews identify associations between heat or pollution exposure and cognitive or psychiatric outcomes in older adults, but observational findings do not establish accelerated neurodegeneration [54]. Cognitive changes during a heat event require assessment for acute medical causes, delirium, medication effects, and environmental barriers.

Children and adolescents may experience climate concern, disruption of education or family routines, and disaster-related distress. Assessment should be developmentally appropriate and distinguish environmental awareness from impairment. School and family support are relevant protective resources, while the youth survey literature does not provide a diagnostic prevalence estimate [43].

People with pre-existing mental disorders may face physical comorbidity, medication-related thermal risk, impaired self-care, or disrupted access to services. A diagnosis does not imply impaired protective behavior in every individual; assessment should consider actual functioning, support, housing, and treatment needs [2,54].

Climate-related hazards can compound existing health inequalities, but the magnitude and pathways vary by setting. Exposure reduction and accessible care should be prioritized using locally measured risk rather than assuming uniform vulnerability within broad population categories [57,58].

Observational cardiovascular and multimorbidity evidence supports attention to older or medically vulnerable groups, while persistent social disadvantage may limit recovery and access to adaptation [42,53,57]. The proposed multisystem framework therefore includes material resources and service continuity as potential determinants rather than treating biology as the sole explanation.

## 8. Clinical Prevention and Adaptation Planning

The practical aim is to reduce exposure and maintain safe, accessible care while identifying people whose distress or psychiatric illness requires additional support. Existing guidance supports integration of mental health into climate action and health-system preparedness; the specific pathway proposed in this review has not been tested as an intervention bundle [59,60].

### 8.1. Contextual Assessment and Clinical Intervention

During heat, disaster, or prolonged environmental disruption, clinicians can ask about exposure, cooling and water access, sleep, medication continuity, substance use, support, and daily functioning. Assessment should include depression, anxiety, trauma symptoms, cognitive change, and suicidal ideation when clinically indicated, followed by established diagnostic and safety procedures. Climate concern itself is not an indication for a psychiatric diagnosis [54,60].

The Climate Change Anxiety Scale and Hogg Eco-Anxiety Scale can characterize climate-specific or broader environmental distress in appropriate language and cultural settings [17,18]. They are not interchangeable and should not be used as stand-alone diagnostic tests or assumed to have universal clinical cutoffs. Scores should be interpreted alongside impairment, baseline mental health, and exposure context [21,22].

Initial support can combine validation of proportionate emotions, practical problem solving, restoration of routines, and connection with family or community resources. Persistent impairment warrants evidence-based care for the identified condition, such as established treatment for depression, anxiety, PTSD, or insomnia. Climate-adapted psychotherapy and collective coping approaches are promising but should not be presented as proven prevention of psychiatric disorders or biological dysregulation [60].

Heat-period care plans should specify hydration and cooling arrangements, medication review, warning signs of heat illness, emergency access, and follow-up for people with substantial clinical or access barriers. Disaster planning should protect prescription continuity and referral routes. After an event, monitoring should consider delayed symptoms and practical recovery needs; blanket compulsory debriefing is not proposed [54,60].

### 8.2. National Adaptation Plans and Heat Action Plans

Figure 3 organizes community support, accessible services, and infrastructure as complementary preparedness domains. It is a policy and implementation framework, distinct from the biological hypotheses in Figure 1 and Figure 2.

National adaptation plans should explicitly include mental health and psychosocial support, identify accountable health and social-care leads, allocate resources, and integrate surveillance and referral into broader climate-health actions. WHO’s mental health policy brief and operational health-system framework support this integration [59,60]. Suggested monitoring indicators include service continuity, time to follow-up after events, unmet care needs, and equitable access to support; these are proposed implementation metrics rather than a validated composite score.

Heat action plans should link early-warning thresholds with targeted outreach, cooling and safe-water access, and coordination between primary care, psychiatric services, pharmacies, emergency departments, and social support. Vulnerability assessment should include clinical functioning, medication-related risks, social isolation, and housing conditions. Communication should be accessible and avoid stigmatizing diagnoses or climate concern [54,59].

Health-system preparedness requires workforce training, resilient premises, backup communication and power, protected medication supply, and continuity plans for community and inpatient mental health care [58,59,61]. Public health practitioners should monitor exposure and service demand, while policymakers should address safe housing, occupational protection, cooling affordability, and equitable adaptation funding. Evaluation must establish whether these measures improve access and outcomes in each setting rather than presume effectiveness.

## 9. Discussion and Research Priorities

### 9.1. Relationship to Existing Frameworks

Palinkas and Wong organize climate-related mental health effects by acute, slower-onset, and ongoing environmental change [13]. Broader climate-health reviews distinguish direct hazards from indirect social and economic consequences [2,4], while emotional frameworks describe threat appraisal, loss, and climate-related affect [15,16]. The present framework retains these distinctions and adds an explicit comparison of biological evidence levels, physical comorbidity, and unequal adaptive capacity; it does not claim that these domains or pathways are newly discovered.

The biological component draws on McEwen’s stress-adaptation and allostatic account [14]. Its extension to climate-related exposure is hypothesis-generating: repeated environmental demands might contribute to impaired recovery, but a persistent multisystem phenotype has not been demonstrated by the reviewed studies. Unlike a deterministic exposure-to-disease chain, the framework allows adaptation, protective resources, and pre-existing conditions to modify trajectories. Exposure associations are strongest where hazards and outcomes are directly measured; biological mediation is less established.

### 9.2. Limitations of Evidence and Review Methods

Eco-distress measures differ in whether they assess worry, affect, rumination, physical symptoms, or functional interference. Existing validated scales improve measurement but do not constitute a common diagnostic standard across cultures and age groups [17,18]. Sampling, translation, construct overlap, and shared self-report methods can affect observed correlations. Associations with symptoms do not establish that environmental awareness represents clinical vulnerability; interpretation requires consideration of functioning, exposure context, cultural meaning, and potentially adaptive engagement [21,22].

Repeated biomarker measurements combined with prospective clinical assessments remain limited. A single cortisol sample cannot establish chronic HPA axis dysregulation, and peripheral inflammatory, BDNF, or GABA measures do not demonstrate central pathology. Exposure misclassification, acclimatization, circadian timing, seasonality, pollution, medication, infection, socioeconomic circumstances, and prior psychiatric illness may affect the findings. Repeated exposure, biomarker, and clinician-assessed outcome measurements are needed to establish temporal ordering and test proposed mediation pathways [3,14,20].

The narrative design introduces potential selection and interpretation bias, language and publication bias, and possible overrepresentation of high-income or well-resourced settings. Section 2 reports the database-specific search strategies and the last search date, 1 July 2026. However, verified database-specific retrieval totals and stage-specific selection counts are unavailable, limiting reproducibility of the retrieval and selection process. Exhaustive coverage cannot therefore be claimed. No formal risk-of-bias assessment was performed, and no pooled estimate was produced. Table 1 provides a representative evidence comparison and should not be interpreted as an exhaustive inventory of eligible studies.

### 9.3. Priorities for Prospective Evaluation

Priority studies should distinguish objective thermal or disaster exposure from perceived threat and include baseline mental health, functioning, medical comorbidity, and adaptive resources. Repeated measures before, during, and after exposure would allow acute effects, recovery, and persistent change to be separated. Clinical endpoints should combine validated symptom measures with structured diagnostic or functional assessments where feasible, rather than biomarker changes alone [20,62].

Mechanistic cohorts should prespecify a small set of endocrine, inflammatory, or redox measures, document sampling conditions, and assess individual exposure with adequate spatial and temporal resolution. Joint exposure models can separate temperature from pollutants and evaluate interaction explicitly. Mediation analyses require temporally ordered data and defensible assumptions; statistical adjustment alone cannot establish a biological pathway.

Intervention studies should evaluate feasible components such as outreach during heat alerts, medication-continuity support, cooling access, and contextual mental health assessment. Pragmatic or cluster designs should compare usual care with clearly defined strategies, assess adverse effects and resource use, and include equity outcomes. The effectiveness of the proposed combined preparedness framework remains unknown [59,60].

Life-course approaches should include youth, older adults, workers, and populations with repeated exposure or disrupted livelihoods [62]. Diverse settings and culturally appropriate measures are necessary because threat appraisal, grief, resources, and service access may differ substantially. Research should measure resilience and recovery as well as impairment.

## 10. Conclusions

Climate-related thermal exposure, disasters, and environmental disruption are associated with adverse mental and physical outcomes, with substantial variation across individuals and settings. Climate anxiety, broader eco-distress, and ecological grief describe meaningful responses to environmental threat or loss and should not be equated automatically with psychiatric disorders. The proposed multisystem framework links these exposure and psychosocial domains with stress biology while explicitly distinguishing clinical associations, experimental mechanisms, and hypotheses.

Short-term human experiments and preclinical models support biological plausibility involving neuroendocrine, inflammatory/redox, and neuroplastic processes. They do not establish a chronic causal chain from climate change to psychiatric illness. Observational designs, heterogeneous measurement, incomplete review search documentation, and limited repeated biomarker and prospective clinical assessment constrain the strength of the conclusions.

For clinicians, the practical priorities are contextual assessment, treatment of clinically significant symptoms, safe medication management, and continuity of care during heat and disasters. For public health practitioners, they are surveillance, accessible outreach, and coordinated response. For policymakers, they are explicit mental health inclusion in adaptation and heat plans, resilient services, and equitable exposure reduction. These actions are supported by existing care principles and guidance; the proposed integrated model remains unvalidated and requires prospective evaluation.

## Figures and Tables

**Figure 1:**
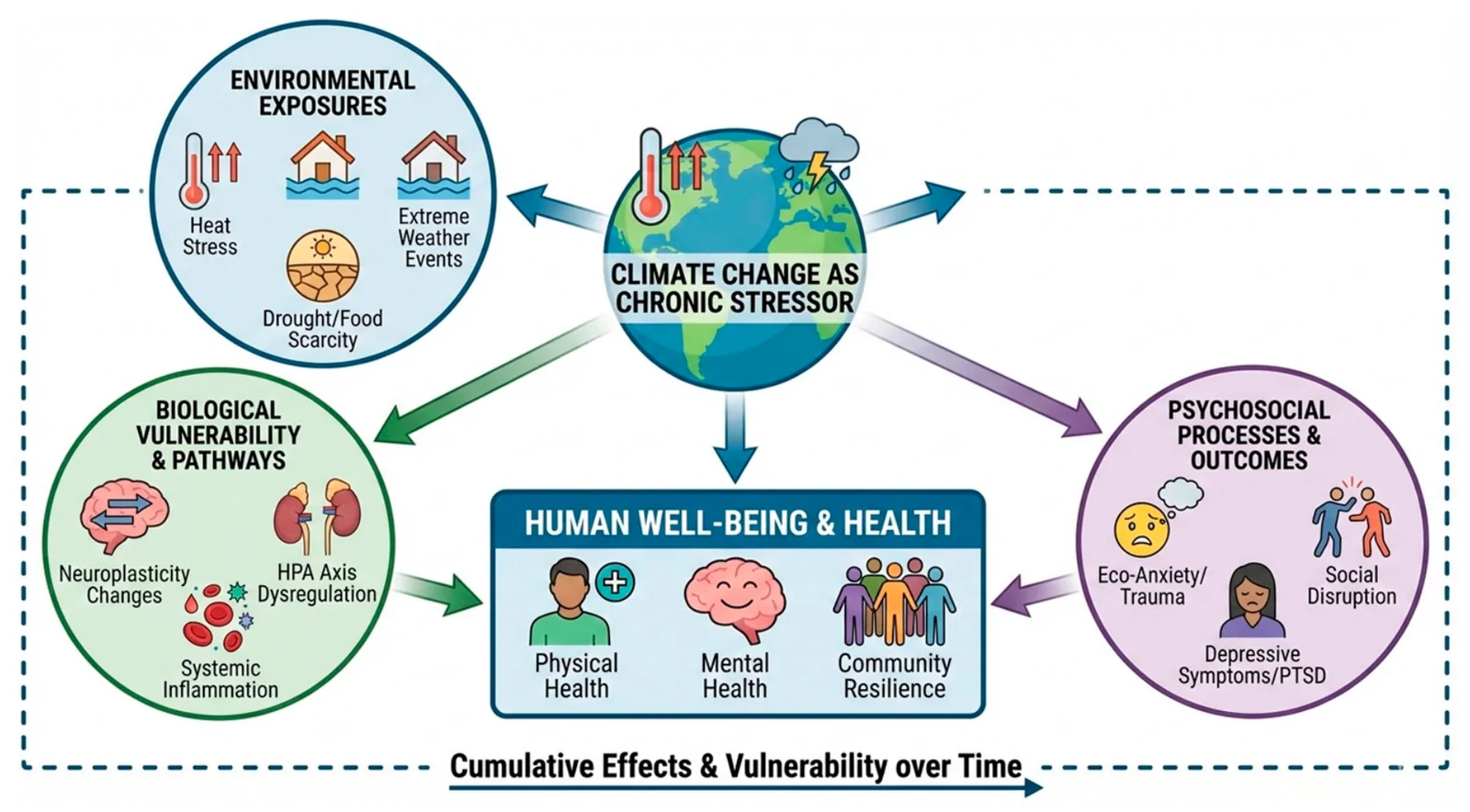
Author-proposed framework relating climate-related exposures, biological susceptibility, psychosocial processes, and human well-being. Note. Environmental hazards, biological susceptibility, psychosocial responses, and health outcomes are shown as interacting domains. Arrows indicate proposed relationships for interpretation and future testing, not uniformly established causal effects or confirmed biological mediation. The framework distinguishes recurrent exposure from emotional responses to environmental threat. Abbreviations: HPA, hypothalamic–pituitary–adrenal; PTSD, post-traumatic stress disorder. The image was generated using ChatGPT-5 (OpenAI) based on instructions provided by the authors. All authors reviewed and approved the image.

**Figure 2:**
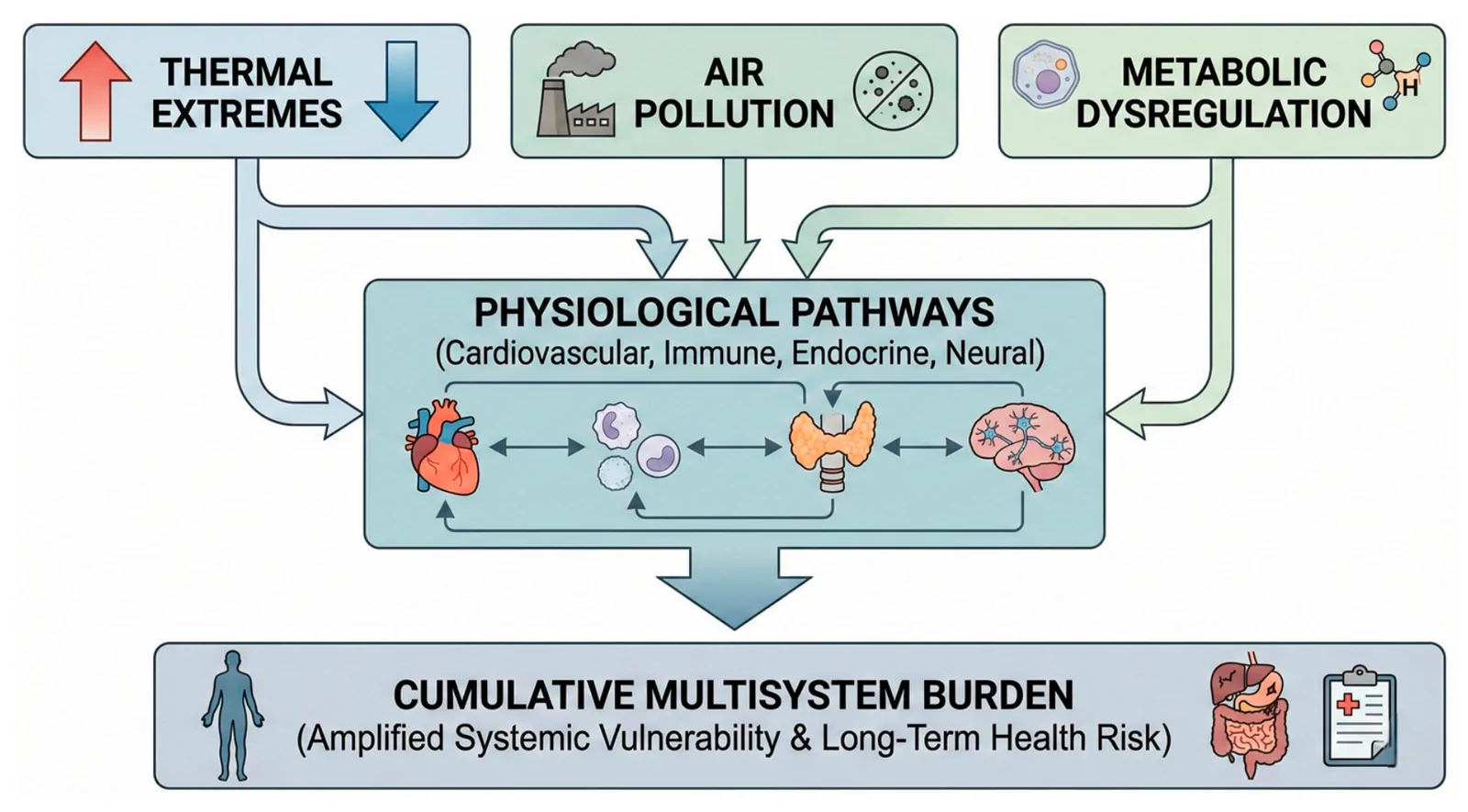
Proposed interactions among thermal exposures, air pollution, physical susceptibility, and multisystem burden. Note. This conceptual figure focuses on physical comorbidity and physiological interactions. Metabolic dysregulation may precede exposure or arise within particular pathways; it is not itself a climatic hazard. Arrows depict proposed interactions rather than confirmed synergistic effects, clinical mediation, or a demonstrated progression to multimorbidity. See Table 1 for the evidentiary limits. The image was generated using ChatGPT-5 (OpenAI) based on instructions provided by the authors. All authors reviewed and approved the image.

**Figure 3:**
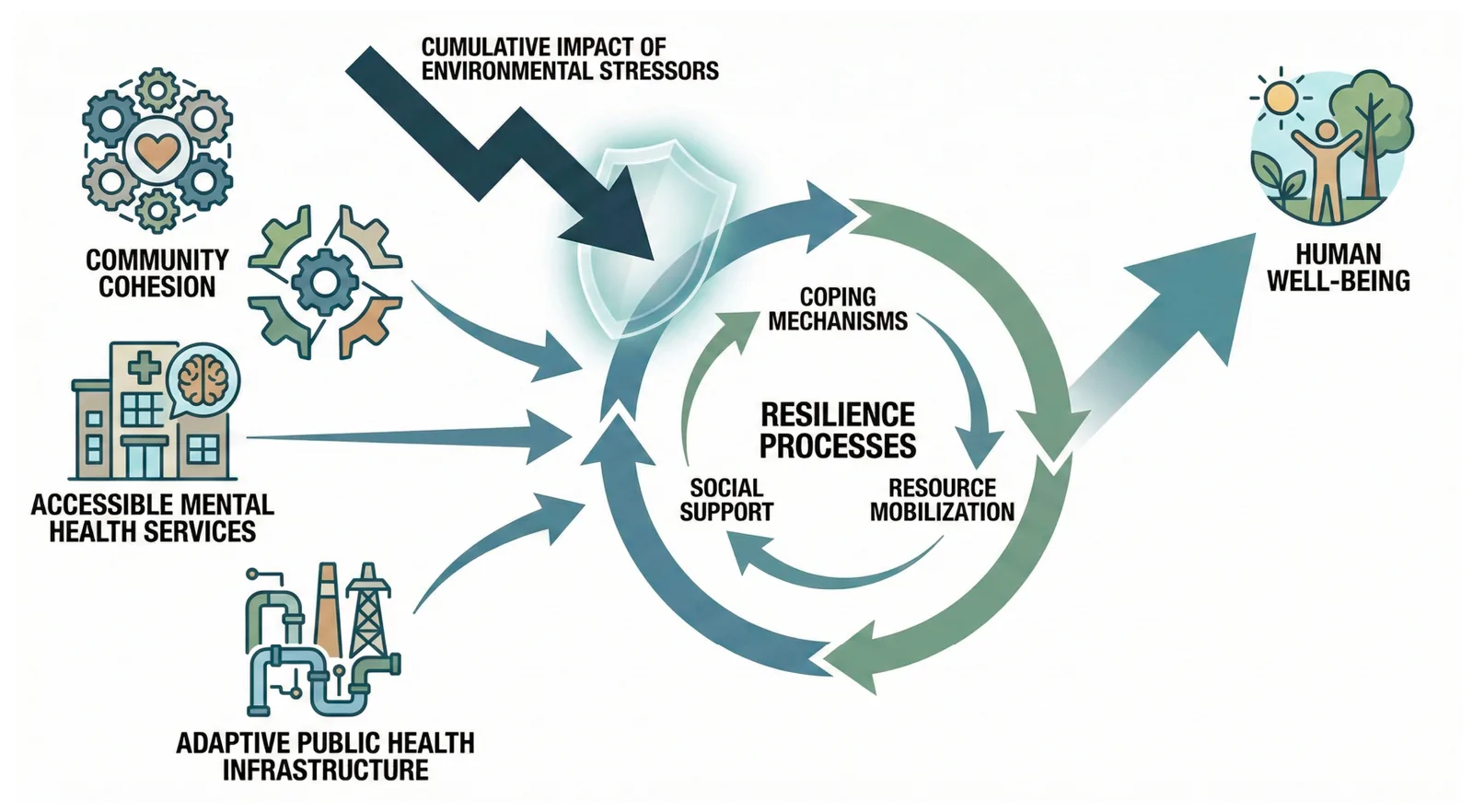
Author-proposed mental health preparedness and community resilience framework. Note. This implementation-oriented framework identifies community cohesion, accessible mental health care, and adaptive infrastructure as potential resources for coping and recovery. Arrows represent planning relationships and proposed protective processes, not demonstrated intervention effects. The framework has not been prospectively validated. The image was generated using ChatGPT-5 (OpenAI) based on instructions provided by the authors. All authors reviewed and approved the image.

**Table 2:** Exposure and vulnerability profiles with proposed assessment and preparedness priorities.

Population or Context	Relevant Exposures and Barriers	Assessment Priorities	Proposed Preparedness Action	Evidence and Limits
Older adults	Heat; isolation; limited cooling; multimorbidity and medication burden	Hydration, cognition, acute medical illness, support and medication safety	Individualized heat plan; accessible cooling; proactive contact and care continuity	Clinical review [54]; risk varies within age groups
Children and adolescents	Disaster disruption; climate uncertainty; school or family instability	Developmental context, sleep, functioning, trauma symptoms and support	Family and school support; age-appropriate communication; referral for impairment	Youth survey [43]; concern does not establish a disorder
Pre-existing mental disorders	Heat; treatment interruption; physical illness or impaired self-care	Actual functioning, prescriptions, thermal medication risk and safe follow-up	Clinician-led medication review; prescription continuity; coordinated outreach	Reviews [2,54]; diagnosis alone is not a risk classifier
Low-income or housing-insecure communities	Heat/pollution co-exposure; unaffordable cooling; transport or care barriers	Objective exposure, safe housing, water, care access and livelihood loss	Affordable cooling and housing measures; accessible local services	Equity and system guidance [57,58,59]; benefit distribution requires evaluation
LMICs or resource-constrained settings	Heat, drought, food/water insecurity and limited service capacity	Locally relevant clinical burden, essential medicines and referral capacity	Integrate psychosocial support with primary care and adaptation planning	Physical-health evidence [55] and policy guidance [59,60]; contextual transferability limited

Note. Priorities are based on existing clinical principles and climate-health guidance; the combined profiles have not been prospectively validated as a screening or prognostic tool. Group membership alone should not determine clinical risk. Abbreviations: LMICs, low- and middle-income countries.

## Data Availability

No new data were created or analyzed in this study. Data sharing is not applicable to this article.
